# A higher frequency of physical activity is associated with reduced rates of SARS-CoV-2 infection

**DOI:** 10.1080/13814788.2022.2138855

**Published:** 2022-11-07

**Authors:** Ilan Green, Eugene Merzon, Shlomo Vinker, Avivit Golan-Cohen, Ariel Israel, Mickey Scheinowitz, Reuven Ishai, Shai Ashkenazi, Eli Magen

**Affiliations:** a Leumit Health Services, Tel Aviv, Israel; b Department of Family Medicine, Sackler Faculty of Medicine, Tel Aviv University, Tel Aviv, Israel; c The Adelson School of Medicine, Ariel University, Ariel, Israel; d Department of Biomedical Engineering, Faculty of Engineering, Tel-Aviv University, Tel-Aviv, Israel; e Sylvan Adams Sports Institute, School of Public Health, Sackler Faculty of Medicine, Tel-Aviv University, Tel-Aviv, Israel; f Neufeld Cardiac Research Institute, Sheba Medical Center, Tel-Hashomer, Israel; g Department of Ear, Nose and Throat, and Head & Surgery, Rambam Medical Center, Haifa, Israel; h Faculty of Medicine, Technion Israel Institute of Technology, Haifa, Israel; i Medicine C Department, Clinical Immunology and Allergy Division, Barzilai University Medical Center, Ben-Gurion University of the Negev, Ashkelon, Israel

**Keywords:** Physical activity, SARS-CoV-2, prevention, health benefits, corona

## Abstract

**Background:**

Physical activity (PA) is associated with health benefits. Previous studies have shown that regular PA decreases the incidence of viral respiratory tract infections, but data on severe acute respiratory syndrome coronavirus-2 (SARS-CoV-2) infection are unavailable.

**Objectives:**

The objective of this study is to examine the association between PA frequency and SARS-CoV-2 infection.

**Methods:**

A population-based cross-sectional study was conducted on data from 1 February 2020 to 31 December 2020, using the registry of Leumit Health Services (LHS), a national health maintenance organisation in Israel. All LHS patients aged 18 to 80 years who underwent at least one RT-PCR test for SARS-CoV-2 during the study period were included. We examined the association between PA frequency (hours per week) and being tested positive for SARS-CoV-2.

**Results:**

Of 113,075 subjects tested for SARS-CoV-2 by RT-PCR (mean age 41.6 years, 54.4% female), 17,465 (15%) were positive. In the SARS-CoV-2-negative group, significantly more subjects were engaged with PA than in the SARS-CoV-2-positive group [crude odds ratio (OR) for any PA 0.75 (95% confidence interval (CI) 0.72–0.77)]. After adjusting for possible confounders, PA frequency had a significant negative association with the likelihood of being SARS-CoV-2 positive (adjusted OR 0.67, 95% CI 0.64–0.68). Moreover, as the frequency of PA increased, the ORs of being SARS-CoV-2-positive decreased (occasional PA: OR 0.71, 95% CI 0.67–0.74; PA 1–3 times/week: OR 0.62, 95% CI 0.58–0.65 and PA > 3 times/week: OR 0.54, 95% CI 0.49 − 0.59).

**Conclusion:**

Our large population-based study in patients undergoing SARS-CoV-2 RT-PCR testing showed that a higher frequency of PA is associated with a lower rate of positive test results

KEY MESSAGES

- Individuals who engage with PA were less susceptible to SARS-CoV-2 infection.
- As the frequency of PA increased, the risk of being SARS-CoV-2-positive decreased.
- PA may have a possible protective effect against viral infections.

## Introduction

Regular physical activity (PA) is associated with many short- and long-term functional, physical, cognitive, clinical and mental health benefits in both children and adults and with reduced mortality [1,2]. Healthcare organisations have, therefore, published national and international evidence-based guidelines encouraging PA [3]. Most guidelines concur that 150-minute PA a week is sufficient for health benefits and for reducing all-cause mortality by 75% and cardiovascular-related mortality by 50% [3,4].

In December 2019, the Coronavirus Disease-2019 (COVID-19) outbreak, caused by the severe acute respiratory syndrome coronavirus-2 (SARS-CoV-2), was reported in China and became a worldwide pandemic [5]. Nearly 80% of the infected individuals are asymptomatic or suffer from mild symptoms, such as cough, coryza and weakness, while few – mainly the elderly and those with underlying medical conditions – can develop severe disease with severe respiratory manifestations, neurological impairment and multi-organ failure [6]. Physical distancing and isolation were adapted to halt SARS-CoV-2 transmission, with quarantines during peaks of the pandemic. Health clubs and sport centres were thus closed for prolonged periods.

The presentation of SARS-CoV-2 infection depends on the interplay between the virus and the host immune system [7]. PA stimulates the immune system, which is needed to reduce the morbidity of SARS-CoV-2 infection and its mental and physical consequences [8,9]. It has been documented that moderate to vigorous PA reduces the risk of respiratory tract infection [10]. Other studies documented augmentation of humoral immune response to influenza vaccination in older adults [11]. Data on PA and SARS-CoV-2 infection are not established [12].

The purpose of this population-based study was to explore the association between PA and the rates of SARS-CoV-2 infection. The study’s specific aims were (1) to evaluate the association of dichotomous PA with SARS-CoV-2 infection and (2) to examine the influence of categories of PA frequency on the rates of SARS-CoV-2-positivity. To explore these associations, we performed bivariable and multivariable analyses.

## Methods

### Study design

We conducted a population-based cross-sectional study utilising data from a large health maintenance organisation, Leumit Health Services (LHS), which provides primary and secondary care services to around 715,000 members dispersed in Israel; acceptance is without any selection process. LHS has a comprehensive computerised database incorporating demographics, medical diagnoses, primary and secondary care medical visits with detailed clinical data results, hospitalisations and laboratory tests. All LHS members hold similar health insurance and equal access to healthcare services. A diagnosis is entered or updated during each physician visit according to the International Classification of Diseases 9th revision (ICD-9). LHS has its own laboratories. Hence, the computerised system automatically incorporates all laboratory results and vaccinations into the patients’ file. There is an ongoing process of data validation, for example by the physicians who are encouraged to report on patients, who in their opinion do not meet the criteria for certain diagnoses. As such, the validity of diagnoses entered into the registry is high for important medical diagnoses, particularly those based on laboratory data [13–15].

The current study was approved by LHS Institutional Ethics Committee (approval number 129-20–LEU).

### Study population

The study period was from 1 February 2020 to 31 December 2020 (the first COVID-19 patients were diagnosed in Israel in February 2020). The study population included all LHS members aged ≥ 18 years who underwent at least one RT-PCR test for SARS-CoV-2 during the study period. Testing was performed by physician’s referral, according to the Israeli Ministry of Health criteria for SARS-CoV-2 testing, which included: direct exposure to a SARS-CoV-2-confirmed individual or presenting symptoms suggestive of COVID-19 (mainly fever with respiratory symptoms such as cough, coryza or shortness of breath) [16,17].

Patients suffering from severe somatic diseases such as congestive heart failure, dementia, hepatic, lung and renal diseases and cancer who are usually incapable of performing PA were excluded. The reason for exclusion was to reduce the risk of bias.

### SARS-CoV-2 testing

Nasopharyngeal swabs were taken and examined for SARS-CoV-2 by a real-time RT-PCR assay with internal positive and negative controls according to the guidelines of the World Health Organization [18]. The SARS-CoV-2 PCR tests used were the 2019-nCoV RT-PCR diagnostic panel Allplex^TM^ 2019-nCoV Assay (Seegene Inc., Seoul, Republic of Korea) or the COBAS SARS-Cov-2 6800/8800 (Roche Pharmaceuticals, Switzerland). COVID-19 RT-PCR tests on nasopharyngeal swabs were performed by experienced personnel in the centralised laboratory of LHS.

### Frequency of PA

Patients are routinely asked during each family physician visit to report information about regular pattern of their PA, including what type and how often. The family physician could mark one of three categories of PA frequency: no PA, PA 1–3 times per week, PA more than three times per week. The PA practices of each enrolee were available in the LHS registry.

### Other variables

Data on demographics [age, gender and socioeconomic status (SES)], smoking status, BMI, certain comorbidities [diabetes mellitus, hypertension and attention deficit hyperactivity disorder (ADHD), selected according to their association with SARS-CoV-2 infection [19–23], and vitamin D level were extracted from the LHS electronic medical records. SES has been applied from the Israeli Central Bureau of Statistics classification system that includes 20 subgroups delineated according to patients’ home address [13]. Classifications 1–7 were defined as low SES, 8–20 middle-high SES.

### Statistical analysis

Statistical analyses were conducted using Statistical Package STATA 12 software (StataCorp LP, College Station, TX, USA).

#### Study population according to SARS-CoV-2 test status

Differences in demographics and comorbidities between the subjects with negative and positive SARS-CoV-2 RT-PCR tests were analysed using Student’s *t*-test and Fisher’s exact *χ*^2^ test for continuous and categorical variables, respectively, based on the normal distribution and variable characteristics. Categorical data is presented in numbers and percentages. Data on continuous variables with normal distribution were presented as mean and standard deviation. We applied multiple imputations for missing data, assuming that data were missing at random conditional on the observed data.

#### Association between SARS-CoV-2 test status and PA frequency

Preliminary evaluation of the association between the risk SARS-CoV-2 positivity and the level of PA was conducted by stratified analyses. Subsequently, multivariable logistic regression analysis adjusted for demographic and comorbidities was applied to estimate the odds ratios (ORs) and 95% confidence interval (CI) for the independent association between SARS-CoV-2 positivity and the level of PA. Adjustment was based on age, gender, smoking status, obesity, vitamin D level and the presence of the comorbidities mentioned above. Multicollinearity of the independent variables was checked by calculating the Variance Inflation Factor (VIF).

## Results

### Study population: demographic characteristics and SARS-CoV-2 positivity

Of the 113,075 subjects (mean age 41.6 years, 54.4% females) tested for SARS-CoV-2, 17,465 (15.4%) were positive. Compared with SARS-CoV-2-negative, SARS-CoV-2-positive individuals were younger (40.2 ± 16.5 vs. 41.8 ± 16.4 years, *p* < 0.001), had a higher proportion of males (8645 (49.5%) vs. 42,930 (44.9%)], *p* < 0.0001], more often belonged to lower SES [13,138 (75.2%) vs. 58,314 (60.9%), *p* < 0.0001] and had a higher BMI (27.1 ± 5.8 vs. 26.8 ± 5.6 kg/m^2^). The SARS-CoV-2-negative group was more likely to be current cigarette smokers [18,940 (19.8%) vs. 1,643 (9.4%), *p* < 0.001].

### The association between PA and SARS-CoV-2 positivity

PA and its frequency were related to SARS-CoV-2 infection rates (Table 1). More SARS-CoV-2-positive than SARS-CoV-2-negative subjects answered that they were not engaged with PA [6907 (39.5%) vs. 31,396 (32.8%), *p* < 0.001]. The presentation of the various levels of PA in the SARS-CoV-2-negative vs the SARS-CoV-2-positive group were occasional PA: 33,125 (34.6%) vs. 6173 (35.3%); PA 1–3 times/week: 21,212 (22.1%) vs. 3118 (17.8%) and PA >3 times/week: 9877 (10.3%) vs. 1267 (7.2%), *p* < 0.001.

**Table 1.** Demographic and clinical characteristics of the subjects by SARS-CoV-2 results.^a^

Variables	SARS-CoV-2 positive (*N* = 17,465)	SARS-CoV-2 negative (*N* = 95,610)	*p* Value
Age (years)	40.2 ± 16.5	41.8 ± 16.4	<0.001
Age category (years)			
<21	2004 (11.4%)	7450 (7.8%)	<0.001
21–40	7474 (42.7%)	42,072 (44.0%)	
41–60	5458 (31.2%)	29,965 (31.3%)	
≥61	2529 (14.4%)	16,123(16.8%)	
Gender			
Male	8645 (49.5%)	42,930 (44.9%)	<0.001
Female	8820 (50.5%)	52,680 (55.1%)	
Socioeconomic status			
Low	13,138 (75.2%)	58,314 (60.9%)	<0.001
Middle–High	4327 (24.7%)	37,296 (39.0%)	
Smoking			
No	13,328 (76.3%)	66,826 (69.8%)	<0.001
Current	1,643 (9.4%)	18,940 (19.8%)	
Past	2494 (14.2%)	9844 (10.3%)	
Physical activity frequency			
Any	10,558 (60.4%)	64,214 (67.2%)	<0.001
Occasionally	6173 (35.3%)	33,125 (34.6%)	
1–3 times a week	3118 (17.8%)	21,212 (22.1%)	
>3 times a week	1267 (7.2%)	9877 (10.3%)	
BMI (kg/m^2^)	27.1 ± 5.8	26.8 ± 5.6	<0.001
Diabetes mellitus	2422 (13.8%)	14,252 (14.9%)	<0.001
Hypertension	2771 (15.8%)	17,804 (18.6%)	<0.001
ADHD	2097 (12.0%)	8535 (8.9%)	<0.001
Vitamin D level (ng/mL)	18.8 ± 9.0	21.0 ± 9.3	<0.001

^a^Data are presented as number (%) or mean ± standard deviation.

ADHD: attention deficit hyperactivity disorder.

Additional variables are described in Table 1. Of note, the SARS-CoV-2-negative group had higher levels of plasma 25(OH) D_3_ than the SARS-CoV-2-positive group (21.0 ± 9.3 vs. 18.8 ± 9.0 ng/mL; *p* < 0.001).

### Multivariable analysis

After adjustment for other variables, male gender, low SES, obesity, ADHD and vitamin D deficiency had a significant positive association with a likelihood for SARS-CoV-2 infection (Table 2). PA had a strong and significant negative association with the likelihood of being SARS-CoV-2 positive. Moreover, as the frequency of PA increased, the ORs of being SARS-CoV-2 positive decreased (occasional PA OR 0.71, 95% CI 0.67–0.74, *p* < 0.001; PA 1–3 times/week OR 0.615, 95% CI 0.579–0.652; PA > 3 times/week OR 0.541, 95% CI 0.496–0.591, *p* < 0.001) (Table 3).

**Table 2.** Crude and adjusted odds ratios (ORs) and 95% confidence intervals (CIs) of variables significantly associated with SARS-CoV-2 positivity.

	Crude OR			Adjusted OR			VIF when all covariates are in the model
Variable	OR	95% CI	*p* Value	OR	95% CI	*p* Value	
Age category (years)							
<21	1.00 (ref.)			1.00 (ref.)			1.13
21–40	0.66	0.62–0.69	<0.001	0.78	0.72–1.00	0.124	
41–60	0.67	0.63–0.71	<0.001	0.92	0.84–1.01	0.121	
≥61	0.58	0.54–0.62	<0.001	0.85	0.77–1.00	0.065	
Male gender	1.20	1.16–1.24	<0.001	1.41	1.34–1.47	<0.001	3.79
Low socio-economic status	1.94	1.87–2.01	<0.001	1.90	1.81–1.99	<0.001	2.05
Smoking							
No	1.00			1.00			3.16
Current	0.43	0.41–0.45	<0.001	0.41	0.38–0.44	<0.001	
Past	1.27	1.21–1.33	<0.001	1.11	1.03-1.20	0.003	
Diabetes mellitus	0.92	0.88–0.96	<0.001	0.99	0.93–1.06	0.835	2.11
Hypertension	0.82	0.78–0.86	<0.001	0.86	0.81–0.92	<0.001	2.31
ADHD	1.39	1.32–1.46	<0.0001	1.48	1.37–1.59	<0.001	2.56
Obesity	1.13	1.09–1.18	<0.001	1.13	1.08–1.18	<0.001	2.57
Low vitamin D^a^	1.46	1.37–1.56	<0.001	1.23	1.15–1.31	<0.001	1.32

^a^Low plasma vitamin D level, was considered as a total plasma 25(OH)D_3_ concentration < 30 ng/mL (75 nmol/L).

VIF: variance inflation factor; ADHD: attention deficit hyperactivity disorder; Obesity, body mass index (BMI) ≥ 30 kg/m^2^.

**Table 3.** Crude and adjusted odds ratios (ORs) and 95% confidence intervals (CIs) for association between physical activity and SARS-CoV-2 positivity.

	Crude OR			Adjusted OR		
Variable	OR	95% CI	*p* Value	OR	95% CI	*p* Value
Physical activity (yes/no)	0.75	0.72–0.77	<0.0001	0.67	0.64–0.68	<0.0001
Physical activity intensity						
No physical activity	1.00 (ref.)			1.00 (ref.)		
0ccasional	0.84	0.81–0.87	<0.0001	0.71	0.67–0.74	<0.001
1–3 times/week	0.66	0.63–0.69	<0.0001	0.62	0.57–0.65	<0.001
>3 times/week	0.58	0.54–0.62	<0.0001	0.54	0.49–0.59	<0.001

Rates of missing data were generally similar for SARS-CoV-2-positive and for SARS-CoV-2-negative subjects; missing data for BMI and laboratory data were less than 10%. The highest rate of missing data was for smoking status (23% of adherent and 25% of non-adherent).

## Discussion

### Main findings

The results of this study clearly indicate that individuals engaged in regular PA are less frequently infected with SARS-CoV-2 than those who are not. The frequency of the activity was also important: as the frequency of PA increased, the rate of SARS-CoV-2 infection decreased. After controlling for other variables known for their association with SARS-CoV-2 infection rates using a multivariate regression analysis, the negative association between PA and the probability of SARS-CoV-2 infection remains independent and statistically significant.

### Strengths and limitations

The present study had several limitations. First, we based our analyses on the self-reported levels of PA. It is known that this type of reporting may be less accurate than the directly observed levels of PA [24,25], as self-reported data have some limitations in terms of their reliability and validity [26]. Nevertheless, the same reporting method was used in both the SARS-CoV-2-positive and negative groups, which included high numbers. Prospective directly observed measurements of PA are nearly impossible in this type of large epidemiologic settings. Second, the type of PA and its intensity can influence the protective effect of PA. Still, these were not encoded in the electronic medical record and thus not available for the study. Third, our inability to discriminate between asymptomatic, symptomatic and severe patients (hospitalisation, intensive care admission) limited evaluating the association between PA and the severity of COVID-19 infection. Fourth, as this was an observational study; we did not investigate the immunological and metabolic mechanisms related to the observed beneficial effects of PA on SARS-CoV-2 infection rate. We therefore used the term association, not causality.

The study’s main strengths were its large size including national data, and its real-world, population-based nature. An additional strength is the inclusion of a multitude of variables that may affect the likelihood for infection with SARS-CoV-2.

### Possible explanations

There might be several explanations for the observed possible protective role of PA on SARS-CoV-2 infection. The main theme is the beneficial effects of PA on both innate and adaptive immune systems that play a significant role in preventing SARS-CoV-2 infection, improving its outcomes, and most importantly, in protecting the vulnerable populations with underlying chronic medical comorbidities from a very severe or even fatal course [27]. During PA, the activity of T lymphocytes, NK cells, B-lymphocytes and monocytes increases, while inflammatory responses and stress hormone levels decrease, leading to an enhancement of immune vigilance; it is plausible that these immune mechanisms can protect against SARS-CoV-2 infection [28,29]. Likewise, numerous prospective studies have reliably shown that PA decreases the risk of both active and latent viral infections [30–32] and the involved mechanisms [33–36].

### Implications

The results of the present study rationalise the desirable public health efforts to educate the community and provide a scientific ground for applying wide-scale PA promotion policies during the COVID-19 pandemic. Additional studies from other institutions and locations, as well as prospective ones, are recommended to repeat our findings and elucidate the mechanisms involved and adopt the findings in public policy statements and in global and national guidelines.

## Conclusion

The present study documents that engagement in PA is associated with reduced rates of SARS-CoV-2 infection and that the association’s magnitude relates to the PA’s frequency. These findings might have implications for public health policies and recommendations.
